# BABA-primed defense responses to *Phytophthora infestans* in the next vegetative progeny of potato

**DOI:** 10.3389/fpls.2015.00844

**Published:** 2015-10-15

**Authors:** Jolanta Floryszak-Wieczorek, Magdalena Arasimowicz-Jelonek, Dariusz Abramowski

**Affiliations:** ^1^Department of Plant Physiology, Poznan University of Life SciencesPoznan, Poland; ^2^Department of Plant Ecophysiology, Faculty of Biology, Adam Mickiewicz UniversityPoznan, Poland

**Keywords:** next-generation SAR, PR1, *Phytophthora infestans*, late blight, potato defense, priming of defense

## Abstract

The transcript of the *PR1* gene accumulation as an informative marker of systemic acquired resistance (SAR) was analyzed in β-aminobutyric acid (BABA) primed potato in the short-lasting (3 days) and long-lasting (28 days) time periods after induction and in the vegetative descendants of primed plants derived from tubers and from *in vitro* seedlings. BABA pretreatment resulted either in minimal or no *PR1* gene expression, but sequential treatment with BABA followed by virulent *Phytophthora infestans* provided data on the imprint of post-stress information and its duration until fertilization, in the form of an enhanced *PR1* transcript accumulation and a transient increase of basal resistance to the late blight disease. The primed state for defense of the susceptible potato cultivar was transmitted to its vegetative progeny as a potentiated *PR1* mRNA accumulation following challenge inoculation. However, variation was observed between vegetative accessions of the BABA-primed potato genotype in responsiveness to disease. In contrast to plants derived from tubers, potato propagated through *in vitro* seedlings largely lost inducible resistance traits, although itretained primed *PR1* gene expression.

## Introduction

In order to develop novel strategies for biotechnological improvement of plant immunity it is necessary to enhance plant recognition capacities for potential attackers, thus boosting the executive responses of disease resistance in plants. Many valuable genotypes of crop plants possess low basal immunity, generally too weak to effectively prevent disease. However, susceptible plants can alter innate immunity through stimulation of systemic defense responses. Plants trigger systemic acquired resistance (SAR) as a result of a frequently weak and local primary infection, caused by various pathogenic and non-pathogenic microorganisms, which is manifested in an enhanced potential to mount immune responses to subsequent infections (Oostendorp et al., 2001; Prime-A-Plant Group, 2006). Factors boosting crop innate immunity include various synthetic compounds known as SAR inducers (Conrath, 2011). This chemical-based technology with a rich arsenal of new synthetic elicitors may be successfully applied in long-lasting crop protection against biotic stresses (Bektas and Eulgem, 2015). Because SAR inducers can offer disease limitation, without the need to be directly toxic for plant or pathogenic microorganisms, they might be promising, environmentally friendly alternatives to conventional pesticides.

Physiological pre-conditioning for faster and stronger plant protection following challenged inoculation may be realized in the strategy of direct induced responses or be based on the phenomenon of priming (Conrath, 2011; Pastor et al., 2013). In contrast to directly induced hyperergic defense responses, priming preliminarily generates changes in the normoergic defense synchronized with the potential storage of information on previous sensing (Janus et al., 2013). The stress imprint is generally composed of biochemical and epigenetic changes (Bruce et al., 2007). The above-mentioned metabolic adaptations to new environmental conditions do not pertain to changes in DNA sequences, but consist of a modification, reversible by different enzymes of DNA- and histone-related proteins, which affects control of gene transcription activities (Zhang, 2008; van den Burg and Takken, 2009; Fu and Dong, 2013).

An important breakthrough in our knowledge on the long-term post-stress metabolic memory in plants was made in 2012, when three independent research groups, i.e., Luna et al. (2012), Pieterse (2012), Rasmann et al. (2012), and Slaughter et al. (2012), revealed evidence for intergeneration inheritance of SAR in plants in relation to biotic factors. Inducing plant defense is a very complex phenomenon because the final result depends on the effectiveness of the SAR inductor, defense responsiveness of the plant and environmental conditions.

It is generally accepted that β-aminobutyric acid (BABA), a non-protein amino acid, is a potent priming agent of SAR in plants. A high effectiveness of the BABA inducer in enhancing resistance was documented in many other pathogen-plant systems, including also the potato-*Phytophthora infestans* system (Cohen, 2002; Baider and Cohen, 2003; Si-Ammour et al., 2003; Ton and Mauch-Mani, 2004; Hamiduzzaman et al., 2005; Ton et al., 2005; Andreu et al., 2006; Dubreuil-Maurizi et al., 2010; Worrall et al., 2012; Janus et al., 2013).

Potato (*Solanum tuberosum* L.) is the fourth most frequently grown crop plant in the world and after wheat and rice it is the third crop in order of importance for human consumption (Haverkort et al., 2009). In turn, potato late blight caused by an oomycete, *P. infestans* (Mont.) de Bary, is one of the most devastating plant diseases worldwide. Losses caused by *P. infestans* are estimated at approximately 16% of annual world potato production (€5.2 billion per annum) and costs of protection against late blight are estimated at millions of dollars as well. Nowadays, late blight control is based on the use of fungicides, which apart from causing economical losses have a negative impact on the environment. Cultivars Russet Burbank from the USA and Bintje from the Netherlands, both more than 100 years old, are still grown widely because of their superior quality and because growers use chemicals to control late blight (Haverkort et al., 2009). In view of this fact new strategies for biotechnological improvement of plant immunity involving the SAR inducers could provide novel agrochemicals to protect crops from diseases.

In our previous papers it was revealed that among various tested SAR inducers only BABA turned out to be an effective factor stimulating callose apposition and promoting systemic resistance to the pathogen in a potato cultivar (‘Bintje’) susceptible to *P. infestans* (Floryszak-Wieczorek et al., 2012; Janus et al., 2013).

In turn, in the presented study we focused on BABA-primed systemic resistance in the same and the next vegetative progeny of a potato cultivar susceptible to *P. infestans*. We found that the primed potato in the short-lasting (3 days) and long-lasting (28 days) time periods after induction and in its vegetative descendants of primed plants derived from tubers after challenged inoculation with the oomycete pathogen exhibited afaster and stronger *PR1* transcript accumulation and limitation of late blight disease progress.

## Materials and Methods

### Experimental Design for the Generation of Progeny Lines

**FIGURE 1:**
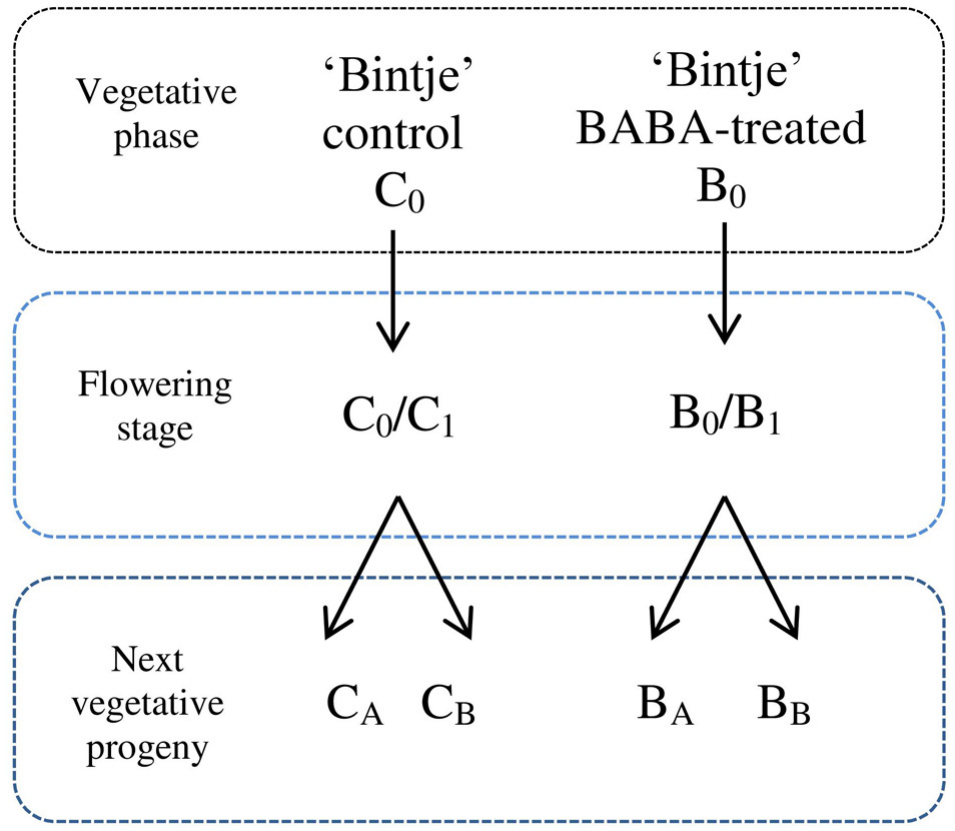
**Experimental design for the generation of progeny lines.** C_0_ – control plant in vegetative phase, B_0_ – plants subjected to immunization with different BABA doses in vegetative phase, C_0_/C_1_ – control plants in flowering stage, B_0_/B_1_ – specimen in flowering stage previously supplied with BABA in B_0_ phase, C_A_ – a batch of healthy plants originating from non-induced C_0_ parents, obtained from lateral buds propagated through *in vitro* seedlings from BABA-treated parental line, C_B_ –regenerated from tubers, B_A_ – plant line generated by *in vitro* micropropagation of BABA-primed parents (in B_0_ phase), B_B_ –regenerated from B_0_ tubers.

Plants were treated with different BABA concentrations as indicated below. Immunization was performed by spraying potato leaves with a selected dose of BABA (3 ml per plant) at the stage of 7–8 leaves of parental plants (B_0_). Some BABA-sensitized plants were inoculated with a *P. infestans* zoospore suspension in order to estimate their immunization level and disease progress. A separate batch of plants was grown until the phase of flowering (B_0_/B_1_; ca. 28 days) and then it was subjected to disease pressure. A fraction of B_0_/B_1_ plants was used as the initial material to obtain vegetative progeny originated in a BABA-sensitized parental specimen. Progeny of induced plants were generated by *in vitro* and *in vivo* propagation, i.e., from lateral buds propagated from *in vitro* seedlings (B_A_) and via tubers (B_B_), respectively. These plants were subsequently cultured to the stage of 7–8 leaves and then inoculated with the oomycete zoospores. A parallel line of non-induced control plants was kept and later exposed to biotic stress to provide a comprehensive comparison of BABA-treated and non-induced plants (C_0_, C_0_/C_1_, C_A_, and C_B_). Plant material was collected for analysis at 24 hpi. Plants exposed to a 1 mM BABA dose were analysed at 1, 3, 6, and 24 hpi. An outline of the experimental design used in the presented paper is given in **Figure 1**.

### Plant Material

A susceptible potato cultivar *S. tuberosum* L. ‘Bintje’ from the Potato Genebank (Plant Breeding and Acclimatization Institute – IHAR-PIB in Bonin) was initially derived from *in vitro* tissue culture and kept in sterile soil in a phytochamber (16 h/8 h : day/night; 180 μmol m^-2^ s^-1^) at 18 ± 1°C and 60% relative humidity.

### Pathogen Culture and Inoculation with *P. infestans*

*Phytophthora infestans* (Mont.) de Bary, virulent for ‘Bintje’ (1.3.4.7.10.11., isolate MP946), was obtained by courtesy from the Plant Breeding and Acclimatization Institute, Research Division in Młochów, Poland. Potato plants were inoculated by spraying leaves with 5 ml of the oomycete zoospore suspension at a concentration of 1.0 × 10^5^ per 1 ml of water and they were kept overnight at 100% relative humidity and 18°C and afterward they were transferred to a growth chamber.

### Immunization with Different BABA Doses

The potato susceptible genotype ‘Bintje’ was immunized by spraying potato leaves with a selected dose of BABA (3 ml per plant; Floryszak-Wieczorek et al., 2012). Concentrations of BABA used in the experiment were as follows: 0.1, 1, 10, and 20 mM and they were delivered to plant surface using an atomizer.

### Assessment of Disease Index

The area affected by disease symptoms was assessed on potato leaves 3–7 days after inoculation with *P. infestans* based on a scale from I to IV (James, 1971), which represented the percentage of leaf area covered by late blight symptoms (I = 1–9%; II = 10–24%; III = 25–49%; IV = 50–100%). Disease symptoms were also determined using trypan blue staining of the *P. infestans* mycelium according to the assay proposed by Wilson and Coffey (1980).

### Gene Expression Measurement

The RNA was isolated from 150 mg of frozen leaf tissue using TriReagent^®^ (Sigma) according to the method of Chomczynski and Sacchi (1987). The obtained RNA was purified with the use of a Deoxyribonuclease I Kit (Sigma). For the reverse transcription 1 μl of RNA from every experimental variant was processed with a RevertAid^TM^ Reverse Transcriptase Kit (Thermo Scientific) according to the manufacturer’s instructions. Real-time PCR was performed on a Rotor Gene 6000 Thermocycler (Corbett Life Sciences). The reaction mixture contained 0.1 μM of each primer, 1 μl of 5× diluted cDNA, 10 μl of the Power SYBR^®^ Green PCR Master mix (Applied Biosystems) and DEPC-treated water to the total volume of 20 μl. The real-time PCR reaction conditions included an initial 5-min denaturation at 95°C, followed by 55 cycles consisting of 10 s at 95°C, 20 s at 53°C and 30 s at 72°C. The reaction was finalized by denaturation at a temperature rising from 72 to 95°C at 1° per 5 s. Reaction specificity was confirmed by the occurrence of one peak in the melting curve analysis.

*PR-1* primers used in real-time detection were as follows:

F: CCGCGTTGAGCTGGGGGAAA, R: GAGCTGGGGACTGCAGGATGC (*T*_m_ = 53°C). The data were normalized to the reference genes encoding the elongation factor (ef1α, AB061263; F: ATTGGAAACGGATATGCTCCA, R: TCCTTACCTGAACGCCTGTCA, *T*_m_ = 53°C) and 18S rRNA (X67238.1, F: GGGCATTCGTATTTCATAGTCAGAG, R: GGTTCTTGATTAATGAAAACATCCT). All used primers were designed using Primer-BLAST (Ye et al., 2012). The *C*_t_ values were determined with the use of a Real-time PCR Miner (Zhao and Fernald, 2005) and the relative gene expression was calculated with the use of efficiency corrected calculation models presented by Pfaﬄ (2004).

### Statistical Analysis

All results were based on at least three independent experiments, each with at least three biological replicates. Analysis of variance was conducted and the least significant differences (LSD) between means were determined using Tukey’s test at the significance level *P* = 0.05. The SigmaPlot 11.0 software (Systat) was used to perform statistical tests. Randomization was performed during collection of samples in the histochemical assay of trypan blue staining.

## Results

In accordance with the experimental design, presented in **Figure 1**, the transcript of *PR1* gene accumulation was analyzed in systemic leaves of BABA primed potato plants in the short (3 days) and long (28 days) time periods after induction and next, in vegetative progeny of primed plants derived from tubers and from lateral buds through *in vitro* seedlings. The listed variants of induced plants were subsequently challenge inoculated with *P. infestans.* All the observed changes were referred to the control, i.e., potato plants not subjected to priming or only inoculated with a virulent pathogen. Moreover, the effectiveness of the applied inducer or priming in the acquisition of systemic resistance in potato plants and in the analysis of inheritance of this trait was assessed on the basis of the disease index assay, i.e. the development of potato late blight symptoms.

### *PR-1* Gene Expression in BABA-Primed Parental Potato Plants (B_0_)

**FIGURE 2:**
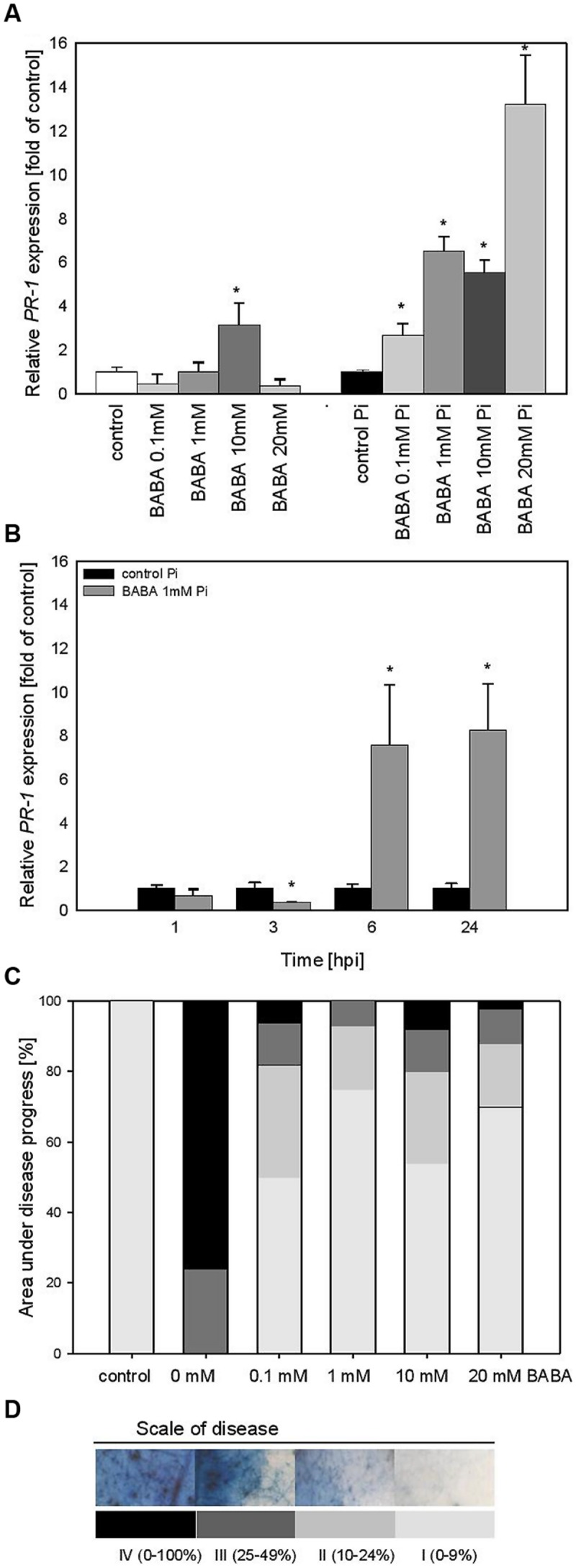
***PR-1* gene transcript accumulation and BABA dose-dependent protection against *Phytophthora infestans* in potato leaves (B_0_). (A)** *PR-1* gene transcript accumulation at 3 days after induction with BABA (left bars) and 24 h after inoculation of previously induced plants (right bars), **(B)** a time-dependent analysis of *PR-1* gene expression in *P. infestans*-inoculated plants previously treated with 1 mM dose of BABA (in B_0_ stage), **(C)** an index of disease development at 7 dpi in directly BABA-treated parents B_0_, **(D)** a scale corresponding to the area of leaves covered with late blight and to trypan blue staining of *P. infestans* mycelium. ^∗^Significantly different from control leaves, *P* < 0.05. Values represent means of data ±SD of at least three independent experiments, each with at least three biological replicates.

The effect of various BABA concentrations on *PR1* transcript accumulation was analyzed 3 days after the plant treatment (B_0_; **Figure 2A**). Generally, the level of *PR1* transcripts was not elevated in plants exposed to BABA, except for a slight rise in the case of the 10 mM inducer dose. In contrast, BABA-sensitized and then challenge inoculated plants exhibited a high rise in *PR1* expression levels from two to sevenfold, depending on the used BABA concentration (**Figure 2B**). Protection of primed potato plants against *P. infestans* was assessed at 7 days post inoculation (**Figure 2C**). The index of disease development in potato leaves represents the percentage of leaf area covered by late blight symptoms, classified into four categories according to the degree of leaf tissue colonization by the pathogen (**Figure 2D**). All applied BABA concentrations exhibited protective effects in terms of disease limitation in comparison to unprimed plants. Because 1 mM BABA-elicited immunity was also effective in plant protection, resulting in approx. 80% disease spot reduction, we focused on this BABA dose when analyzing *PR1* transcript levels at different time points after challenge inoculation. The concern was that the highest concentrations of BABA (10 and 20 mM) could induce hyperergic defense effects or be transferred to the next generation.

Thus, we found that the time course of *PR1* mRNA expression in 1 mM BABA-treated and subsequently inoculated potato leaves showed a 10-fold higher expression level, peaking at 6 and 24 hpi in relation to unprimed and inoculated plants (**Figure 2B**).

### *PR-1* Gene Expression in BABA-primed Parental Potato After its Passage to the Generative Stage (B_0_/B_1_)

**FIGURE 3:**
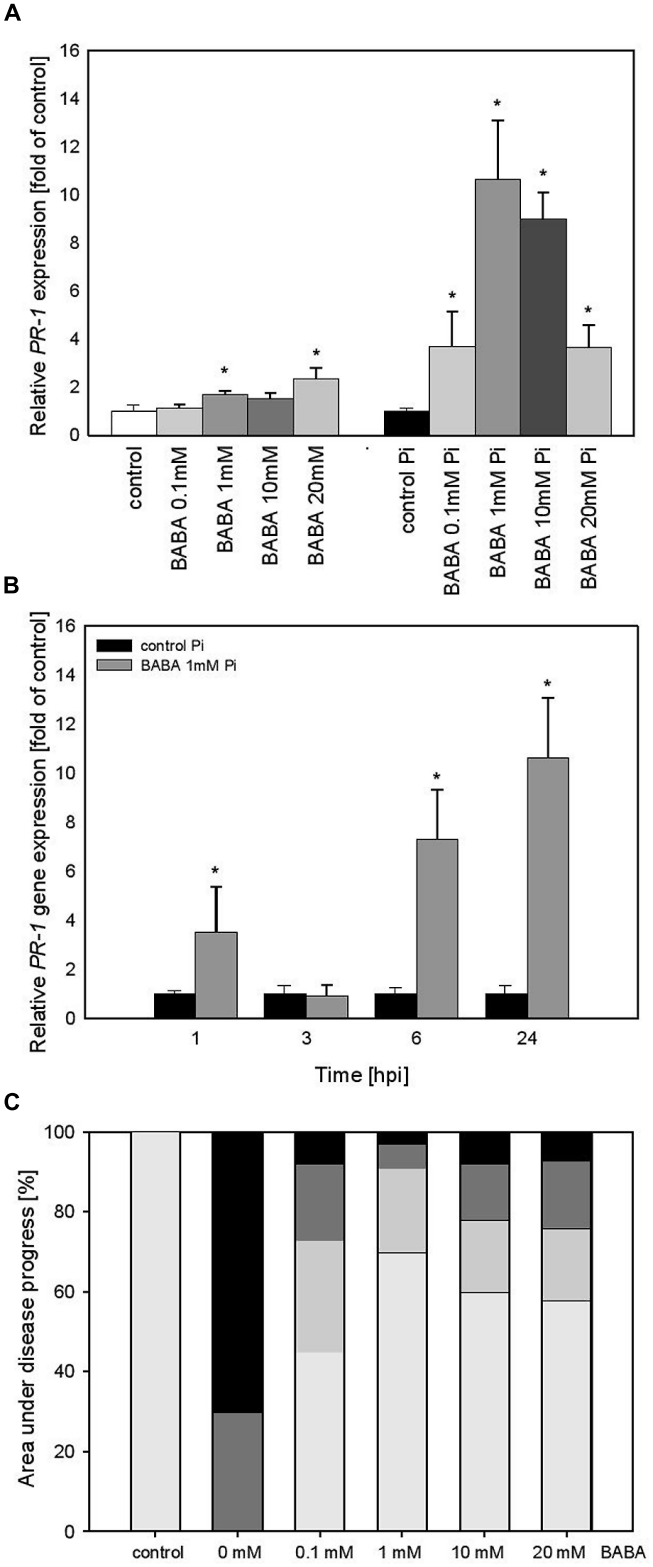
***PR-1* gene transcript accumulation and BABA dose-dependent protection against *P. infestans* in the flowering stage (B_0_/B_1_) of BABA-treated parent plants. (A)** *PR-1* gene transcript accumulation 28 days after induction with BABA (left bars) and after inoculation of previously immunized plants (at 24 hpi; right bars), **(B)** a time-dependent analysis of *PR-1* gene expression in *P. infestans*-inoculated plants previously treated with 1 mM dose of BABA (28 days earlier). **(C)** disease development index at 7 dpi in the BABA-treated parents in flowering stage (B_0_/B_1_). ^∗^Significantly different from control leaves, *P* < 0.05. Values represent the means of data ±SD of at least three independent experiments, each with at least three biological replicates.

To confirm that priming is not reversed after establishment of flower buds by potato plants we performed experiments over a longer time period after BABA-treatment of parental plants. A slightly more intensive *PR1* expression level (ranging from 1.2 to 2.2), correlated with increased BABA concentrations, was found in potato leaves at 28 days (**Figure 3A**) in comparison to 3 days after the treatment (**Figure 2A**). In turn, the BABA-mediated mRNA transcript for *PR1* was rapidly up-regulated after challenge inoculation and – interestingly – it was the most elevated (10-hold higher) in 1 mM BABA supplied plants (**Figure 3A**). An independent analysis of *PR1* time expression patterns revealed that potato plants pretreated with 1 mM BABA showed an earlier expression (since 1 hpi) and a higher transcription abundance (up to 24 hpi) than unprimed plants (**Figure 3B**). Furthermore, BABA-primed potato plants B_0_/B_1_showed enhanced resistance against *P. infestans* compared to C_0_/C_1_ (**Figure 3C**). Plants from this progeny, previously treated with 1 mM BABA, exhibited nearly 70% reduction of late blight symptoms.

### Transcript Accumulation of *PR1* in Vegetative Progeny of BABA-primed Plants Derived from Tubers (B_B_)

**FIGURE 4:**
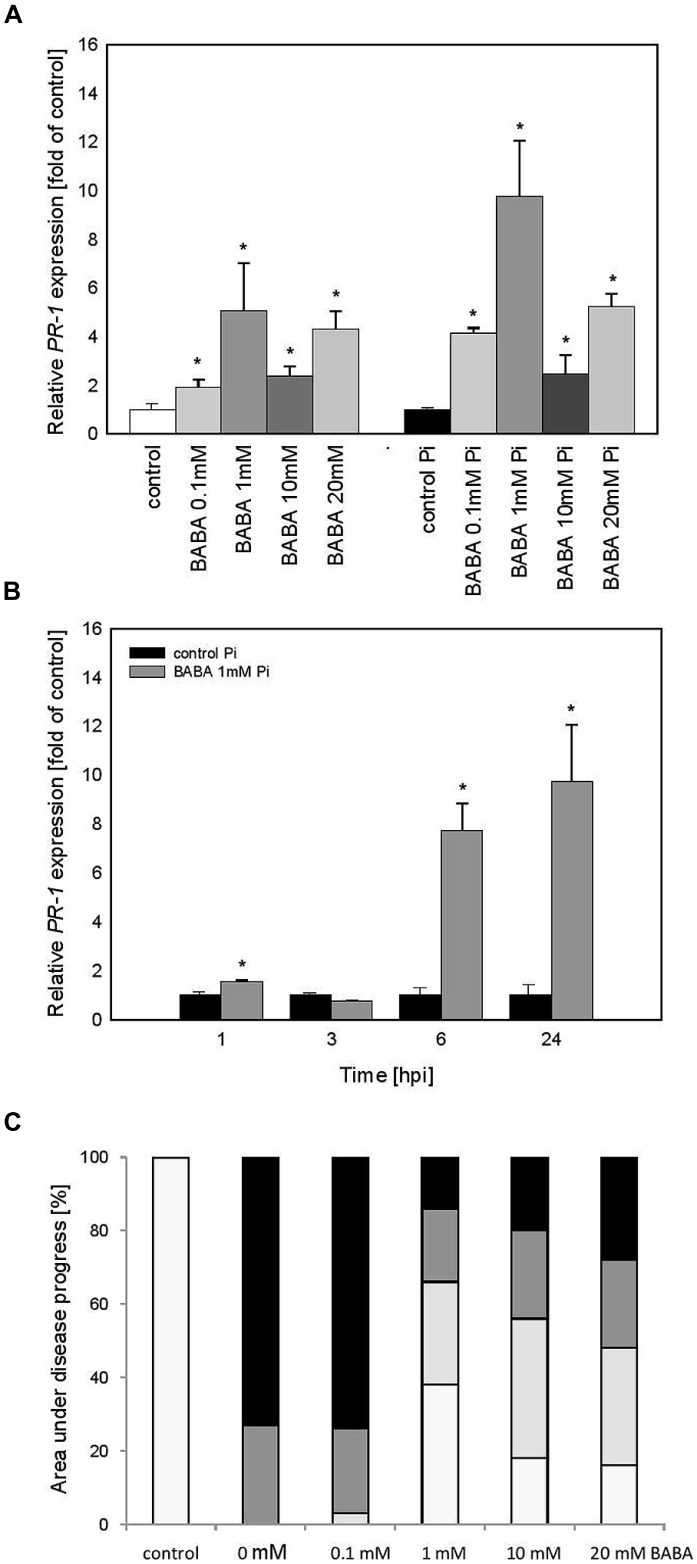
***PR-1* gene transcript accumulation in progeny lines of immunized potato plants (B_B_ stage) -offspring received from the BABA-treated parental line with use of tubers. (A)** After induction of B_0_-originated plants with BABA (left bars) and 24 h after inoculation of previously immunized plants (right bars), **(B)** a time-dependent analysis of *PR-1* gene expression in *P. infestans*-inoculated plants obtained from the tubers of plants exposed to 1 mM BABA (in B_0_ stage), **(C)** disease development index at 7 dpi in the BABA-treated parental line with use of tubers, ^∗^significantly different from control leaves, *P* < 0.05. Values represent means of data ±SD of at least three independent experiments, each with at least three biological replicates.

To examine the persistence of stress memory in the case of acquired resistance we analyzed potato progeny grown from tubers generated from primed plants (B_B_ line). Obtained data revealed a similar tendency in *PR1* gene activation and immunity as it was found in BABA-primed parental plants. Thus vegetative offspring produced from potato tubers showed a slight expression of *PR1* before inoculation and an enhanced *PR1* induction upon potent challenge with the oomycete pathogen (**Figure 4A**). Analysis of progeny of 1 mM BABA primed plants revealed a long-lasting impact on time-dependent kinetics of *PR1* gene expression augmented at 6 and 24 hpi (**Figure 4B**). Moreover, it was documented that primed progeny of induced plants retained an acquired systemic resistance to *P. infestans* in the form of approx. Sixty percentage diminished disease spot area compared to the infected unprimed leaves (**Figure 4C**).

### Transcript Accumulation of *PR1* in Vegetative Progeny of BABA-primed Plants Derived from Lateral Buds Propagated Through *in Vitro* Seedlings (B_A_)

**FIGURE 5:**
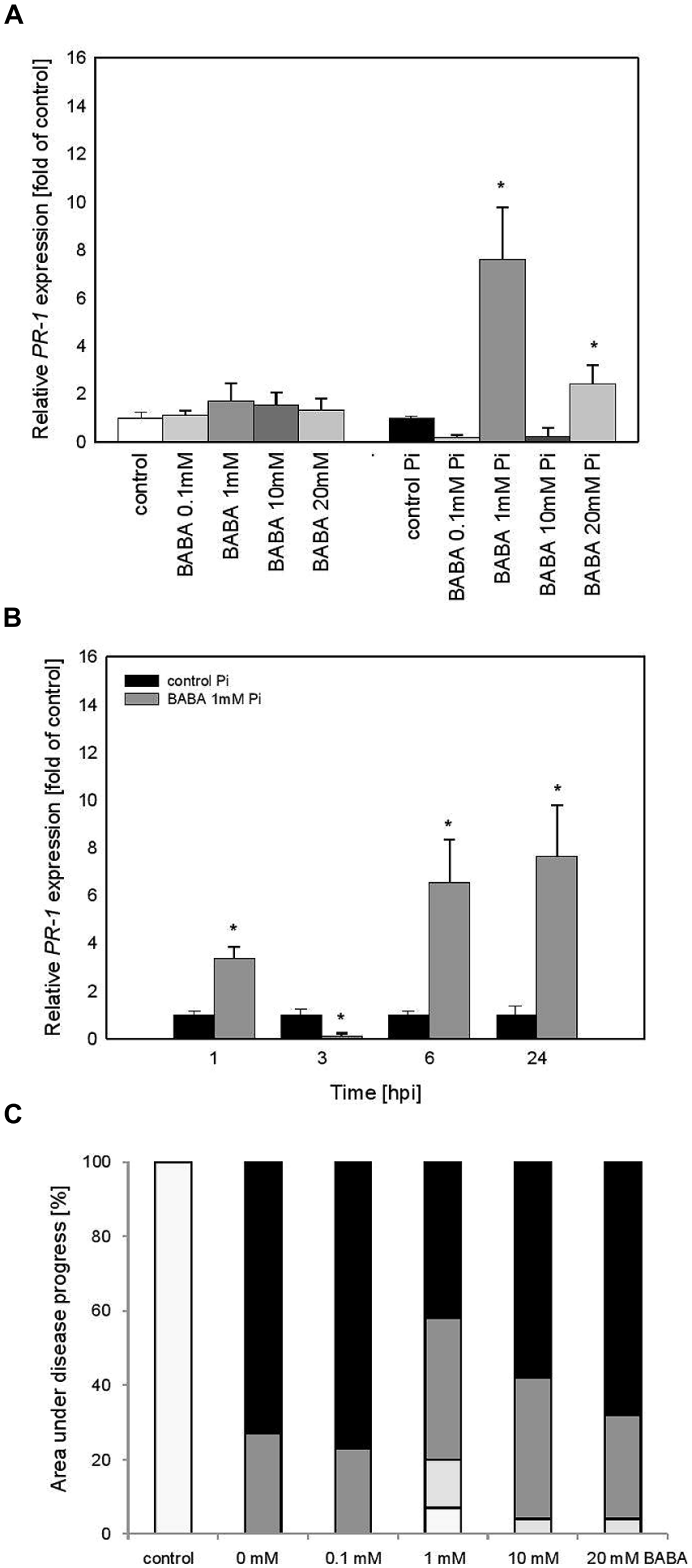
***PR-1* gene transcript accumulation in progeny lines of immunized potato plants (B_A_) – offspring received from the BABA-treated parental line with use of plants derived from lateral buds propagated from *in vitro* seedlings. (A)** After induction of B_0_-originated plants with BABA (left bars) and 24 h after inoculation of previously immunized plants (right bars), **(B)** a time-dependent analysis of *PR-1* gene expression in *P. infestans*-inoculated plants obtained from the parent sensitized with 1 mM BABA (in B_0_ stage). **(C)** disease development index at 7 dpi in the BABA-treated parental line with use of plants derived from lateral buds propagated from *in vitro* seedlings, ^∗^significantly different from control leaves, *P* < 0.05. Values represent means of data ±SD of at least three independent experiments, each with at least three biological replicates.

Vegetative progeny of induced potato plants through *in vitro* culture showed sensitization to *PR1* priming triggered by BABA and thus created a stress imprint activation, facilitating acquisition of a competence to react faster after challenge inoculation. It was reflected in the minimal rise of *PR1* levels before infection and an enhanced induction of the gene expression upon pathogen treatment (**Figure 5A**). Vegetative progeny of 1 mM BABA-primed plants derived from lateral buds (**Figure 5B**) displayed comparable kinetics and slightly less intensity of *PR1* transcript accumulation than those of primed progenies grown from tubers in the successive 24 h after challenge inoculation (**Figure 4B**).

Summing up, the primed state of the susceptible potato cultivar (cv. Bintje) was transferred to its vegetative progeny as a potentiated *PR1* gene expression following challenge inoculation. Nevertheless, the potato plants from *in vitro* cultures have largely lost the trait of acquired resistance to *P. infestans* and the leaf area affected by late blight was similar to that of the infected control (**Figure 5C**).

## Discussion

Results on the sequential potato plant treatment with BABA followed by the virulent *P. infestans* challenge inoculation provided data on the imprint of post-stress information and its long persistence until fertilization, in the form of an enhanced *PR1* transcript accumulation and a transient improvement of acquired resistance to the late blight disease.

A very important issue in evaluating the effectiveness of SAR is connected with the proper selection of informative markers for the trait acquired by the plant and potentially enhanced or inherited. Generally it is known that establishment of SAR is closely related with systemic activation of *pathogenesis-related* (*PR*) genes coding plant defense proteins (Ryals et al., 1996). There is increasing evidence that among various *PR* genes the *PR1* gene is the most responsive to priming in an induced-parental plant and its progeny (Luna and Ton, 2012; Luna et al., 2014b). In priming for defense the key issue is that *PR1* gene expression as a good fingerprint of BABA-induced is mainly switched on after challenge inoculation. Our results showed that both potato plants directly exposed to BABA (B_0_ and B_0_/B_1_) and their vegetative progenies (B_A_ and B_B_) were sensitized to priming of *PR1*, thus they displayed stress memory of the previous treatments, to which their parental plants had been subjected to. Importantly, BABA pretreatment induced either minimal or no *PR1* gene expression, but the metabolic memory of the treatment had to be saved and appeared as the consequence of the pathogen attack. Therefore, transient enhancement of the *PR1* transcript level has been revealed only when the plant was challenged with *P. infestans* in the same generation-primed potato and its next vegetative progeny.

When analyzing the long history of research on SAR in plants it needs to be stressed that research hypotheses proposed in relation to this problem, apart from supporters, have also been questioned by many opponents (Heidel et al., 2004; Walters and Heil, 2007). A frequently quoted argument against SAR was connected with the reservation concerning yield reduction and variation in plant responsiveness to defense elicitors caused by genotype and environment (Bruce, 2014). It was also emphasized that acquired resistance is reversed upon entering the reproductive phase by plants, due to the altered phytohormone balance (Molinier et al., 2006; Pecinka and Scheid, 2012; Banday and Nandi, 2015). Therefore BABA priming efficiency was analyzed by us in potato plants in the flowering state. Obtained data revealed that memory of sensitization had to be retained in potato plants, as in the presence of the pathogen the level of *PR1* increased (10-hold) at 28 days after the BABA treatment.

Jakab et al. (2001) in *Arabidopsis thaliana* showed that BABA applied as a foliar spray, in contrast to soil drench, even at low concentrations, enhanced the accumulation of *PR1* mRNA. Moreover, both experimental approaches led to the induction of resistance, suggesting that in this case the establishment of resistance could be independent of *PR1* expression. Generally speaking, PR1 accumulation is highly dependent on plant genotype and may change significantly under controlled conditions of the phytochamber when compared to the uncontrolled conditions found in the greenhouse or in the field (Cohen et al., 1994; Cohen, 2002). Hence, different mechanisms of protection are effective against distinct pathogens, and BABA can prime the plant to arrange such pathogen-specific responses much faster (Zimmerli et al., 2000).

To study the transmission of the BABA-primed effects to the vegetative potato progenies we observed the persistent state of priming in the form of enhanced *PR1* expression and slowed down development of late blight symptoms on leaves of plant obtained from tubers. Thereby we found that the primed state in the sensitized potato cv. Bintje was preserved over one generation and translated to the descendants propagated through mitotic divisions. Because potato cultivars usually far more resistant to *P. infestans* are commercially propagated by tubers, obtained data may be of great practical importance. Late blight in the field can progress very rapidly and destroy potato foliage within a very short time under favorable weather conditions, causing annual losses from a few to several billion dollars worldwide (Schepers et al., 2009; Hu et al., 2012). BABA-primed resistance might provide new tools to improve crop protection thanks to the enhanced natural defense ability of potato plants to *P. infestans* or the reduced use of fungicide (Liljeroth et al., 2010).

The effectiveness of BABA to induce resistance against *P. infestans* was explored by Olivieri et al. (2009) in potato cultivars differing in their level of resistance to late blight. Obtained tubers from BABA treated plants, challenge inoculated with *P. infestans*, showed a much more pronounced increase in phenol and phytoalexin levels in resistant (cv. Pampeana) rather than in susceptible (cv. Bintje) potato plants, when compared to untreated ones. Likewise, in both the above-mentioned potato cultivars BABA pretreatment improved the yield of harvested tubers. Cultivar-dependent differences in the response to BABA application were also studied by Bengtsson et al. (2014). The results confirmed previous data (Olivieri et al., 2009) that BABA treatment enhanced resistance of potatoes, although the efficiency of BABA supply differs between potato cultivars. Thus, in a more resistant cv. Ontario both a significant reduction in *P. infestans* growth and the activation of various defense responses were faster and stronger than in cv. Bintje (Bengtsson et al., 2014). However, according to these authors the observed changes should be rather attributed to direct defense responses of potato, rather than primed ones.

Our results demonstrate that the primed state for defense of a susceptible potato cultivar (cv. Bintje) is transmitted to its vegetative progeny as a potentiated *PR1* mRNA accumulation after further challenge with *P. infestans*, with protection against pathogen attack in plants derived from tubers. Despite the similarities in the *PR1* transcriptional response, plants derived from lateral buds propagated through *in vitro* seedlings revealed a lesser potential to switch on effective defense pathways to the oomycete pathogen. Generally each BABA-primed genotype, including cv. Bintje, possesses the capacity to mobilize post-stress responses via SA-dependent and/or JA/ethylene–dependent regulatory pathways within its own defense threshold to face the following challenge infection. A relatively low basal resistance of the susceptible cv. Bintje was not sufficient to effectively defend plants of the next primed progeny from *in vitro* against the oomycete pathogen. Apart from the *PR1* gene expression analyzed in our experiment, priming leads to an augmented activation of multi-genic defense mechanisms (Ahmad et al., 2010). It has not been excluded that plant exposure under *in vitro* conditions could affect potato seedling responsiveness.

According to Bruce (2014), induced plant defense is a complex phenomenon and its high variability depends on plant genetics and physiology, while it may also be altered by environmental conditions. Moreover, experiments conducted on 32 tomato accessions using BABA revealed that protection of induced plants against *P. infestans* was not identical on accessions exhibiting the same level of susceptibility and both leaf position and isolate interacted with inducibility (Sharma et al., 2009). In the next experimental approach they confirmed the previous statement that the level of induced defense was not always related to the resistance level of the tomato accession and it was significantly influenced by the pathogen isolate used for challenge inoculation (Sharma et al., 2010). Thus, insight into the molecular basis of priming for defense becomes therefore absolutely essential and will significantly facilitate increased plant basal resistance without negative or compromise effects.

Another significant problem in BABA application as a crop defense activator is connected with the inhibition of plant growth when used in high doses (van Hulten et al., 2006; Walters and Heil, 2007). Recent studies of Luna et al. (2014a) revealed that cellular perception of BABA is mediated by aspartyl-tRNAsynthetase (AspRS) encoded by the *IMPAIRED IN BABA-INDUCED IMMUNITY 1* (*INB1*) gene. They documented the functioning of two separate regulatory pathways managed by *IBI1*, using the *A. thaliana* mutant impaired in BABA-induced disease immunity (*ibi1*), but which was hypersensitive to BABA-induced growth repression. According to the authors, an in-depth clarification of these independent molecular mechanisms opens new possibilities to engineer constitutively primed plants without BABA treatment and growth inhibition (Schwarzenbacher et al., 2014).

The concept of epigenetic control of defense priming has been generally accepted by many research groups; however, more details on this phenomenon will be needed (van den Burg and Takken, 2009; Luna and Ton, 2012; Luna et al., 2014b). We assumed in our experimental approach that obtaining successive vegetative generations of a highly susceptible potato cultivar with the state of increased alertness in the form of enhanced *PR1* expression toward virulent *P. infestans* provides the starting point for the identification of molecular carriers of such inherited post-stress information. It will also be interesting to learn where lies a functional link between the state of readiness and the executive state of plant immunity.

## Conflict of Interest Statement

The authors declare that the research was conducted in the absence of any commercial or financial relationships that could be construed as a potential conflict of interest.
